# A84 SECURE ELECTRONIC DOCUMENT SIGNING UPTAKE IN BIOLOGIC PRESCRIBING FOR IMMUNE MEDIATED DISEASES

**DOI:** 10.1093/jcag/gwab049.083

**Published:** 2022-02-21

**Authors:** S Sharma, J Jones, M Stewart

**Affiliations:** 1 Gastroenterology, Dalhousie University, Halifax, NS, Canada; 2 Medicine, Dalhousie University, Halifax, NS, Canada

## Abstract

**Background:**

The COVID-19 pandemic drastically impacted workflows in gastroenterology practice. Physicians managing immune-mediated diseases (IMDs) must complete special authorization (SA) and prescription renewal (Rx) forms for patients on biologic therapy. This adds significant administrative burden potentially leading to delays in therapy initiation and care continuity. Historically, document completion has largely been paper-based, with forms faxed between patient support programs (PSPs) and physician offices. Disruption of normal office processes during the pandemic necessitated the movement of existing paper-based workflows online. The use of secure electronic document signing (SEDS) platforms has allowed physicians to receive and complete documents digitally.

**Aims:**

To evaluate the impact of SEDS-based biologic documentation on clinical practice. Objectives were 1) to determine if the use of SEDS platforms increased timeliness of document returns compared to traditional workflows 2) assess whether SEDS usage is acceptable and sustainable and 3) assess MD satisfaction with SEDS platforms.

**Methods:**

This was a retrospective audit of SEDS and paper-based biologic document workflows from a single PSP (Abbvie Care). Outcomes of interest were the number of documents completed monthly using SEDS, new monthly users, and the number of active monthly users between April 1, 2020-March 31, 2021. Time (days) to SEDS completion (vs. paper process) was determined by reviewing timepoint data for SA and Rx documents from May 2019-January 2020 (‘pre- SEDS’) and for SEDS documents from May 2020-January 2021(‘SEDS’). The return time (RT) was defined as the time between date sent to a physician’s office by the PSP to the date returned to the PSP. Documents in the pre-SEDS cohort with a RT exceeding 30 days were excluded

**Results:**

In totality, 5573 SA and Rx documents were completed by 383 physicians using the SEDS platform from April 2020-March 2021. A mean of 14.6 (sd 21.8) documents were signed per physician. The number of monthly electronic documents processed increased from 104 in April 2020 to 800 in March 2021. Active monthly users increased from 24 in April 2020 to 213 in March 2021 (31 new users monthly). A total of 19,387 paper documents were processed during the ‘pre-SEDS’ period and 3,317 electronic documents processed in the ‘SEDS’ period. The mean RT in the ‘pre-SEDS’ period was 8.03 days (Sd 8.2) and the ‘SEDS’ period was 1.11 days (sd 2.6).

**Conclusions:**

This data demonstrates acceptability, appropriateness, and improved processing efficiency of a SEDS platform improving timeliness of patient care. Next steps in this research include surveying physicians to understand the work-flow impact of SEDS, functionality, long-term sustainability, satisfaction and impacts on disease related outcomes.

**Funding Agencies:**

None

